# Psychological distress from early adulthood to early old age: evidence from the 1946, 1958 and 1970 British birth cohorts

**DOI:** 10.1017/S003329172000327X

**Published:** 2022-06

**Authors:** Dawid Gondek, David Bann, Praveetha Patalay, Alissa Goodman, Eoin McElroy, Marcus Richards, George B. Ploubidis

**Affiliations:** 1 Centre for Longitudinal Studies, UCL Institute of Education, University College London, London, UK; 2 MRC Unit for Lifelong Health and Ageing at UCL, University College London, London, UK; 3 Department of Neuroscience, Psychology and Behaviour, University of Leicester, Leicester, UK

**Keywords:** Adulthood, BCS70, British birth cohorts, common mental disorders, life course, mental health, NCDS, NSHD, psychological distress, trajectory

## Abstract

**Background:**

Existing evidence on profiles of psychological distress across adulthood uses cross-sectional or longitudinal studies with short observation periods. The objective of this research was to study the profile of psychological distress within the same individuals from early adulthood to early old age across three British birth cohorts.

**Methods:**

We used data from three British birth cohorts: born in 1946 (*n* = 3093), 1958 (*n* = 13 250) and 1970 (*n* = 12 019). The profile of psychological distress – expressed both as probability of being a clinical case or a count of symptoms based on comparable items within and across cohorts – was modelled using the multilevel regression framework.

**Results:**

In both 1958 and 1970 cohorts, there was an initial drop in the probability of being a case between ages 23–26 and 33–34. Subsequently, the predicted probability of being a case increased from 12.5% at age 36 to 19.5% at age 53 in the 1946 cohort; from 8.0% at age 33 to 13.7% at age 42 in the 1958 cohort and from 15.7% at age 34 to 19.7% at age 42 in the 1970 cohort. In the 1946 cohort, there was a drop in the probability of caseness between ages 60–64 and 69 (19.5% *v.* 15.2%). Consistent results were obtained with the continuous version of the outcome.

**Conclusions:**

Across three post-war British birth cohorts midlife appears to be a particularly vulnerable phase for experiencing psychological distress. Understanding the reasons for this will be important for the prevention and management of mental health problems.

## Introduction

Common mental disorders (including depression and anxiety) are the leading cause of non-fatal disease burden, particularly in middle- and high-income countries (Whiteford et al., 2013), and their impact has increased over the last three decades (GBD 2015 Disease and Injury Incidence and Prevalence Collaborators, 2016). Psychological distress captures depressive and anxiety symptoms (Mirowsky & Ross, 2002). Studying age effects on distress can help identify high-risk life periods – with modifiable risk factors – facilitating prevention and early detection of these disorders (Burton-Jeangros, Cullati, Sacker, & Blane, 2015). Cross-cohort comparisons of distress profiles can also elucidate whether risk occurs at consistent phases of the life course, or varies according to changing social and economic circumstances (Sullivan, Brown, & Bann, 2015).

Evidence in the United Kingdom (UK) and other high-income countries has so far relied on repeated cross-sectional or longitudinal studies following individuals over a short period. The most common finding across recent repeated cross-sectional (Blanchflower, 2020; Blanchflower & Oswald, 2008; Spiers et al., 2012) and longitudinal studies in the UK (Bell, 2014; Sacker & Wiggins, 2002) has been an increase in mental health problems between early adulthood and midlife (Bell, 2014; Blanchflower & Oswald, 2008; Sacker & Wiggins, 2002; Spiers et al., 2012), followed by a subsequent decline in early old age (Blanchflower & Oswald, 2008; Spiers et al., 2012). However, there have been inconsistencies in the evidence – with some studies showing an initial decrease in psychological distress from late adolescence to early adulthood and subsequent increase in midlife (Sacker & Wiggins, 2002), and others finding stable levels of distress (Jokela, Batty, & Kivimaki, 2013) or continuous increase from late adolescence to midlife (Bell, 2014; Blanchflower & Oswald, 2008). Similarly, the finding of the apparent decline of psychological distress between midlife and early old age (Blanchflower & Oswald, 2008; Jokela et al., 2013; Keyes et al., 2014) is highly inconsistent – with some studies also showing stable and subsequently worsening mental health in the UK (Bell, 2014).

This study aimed to investigate the age profile of psychological distress in three British birth cohorts – initiated in 1946 (the MRC National Survey of Health and Development; NSHD), 1958 (National Child Development Study; NCDS) and 1970 (the British Cohort Study; BCS70). Combining and comparing these three studies allowed for investigating age and cohort effects on psychological distress among 28 362 participants, aged 23–69, between 1981 and 2016. A comparison across birth cohorts allows for the degree of generalisation of findings across post-WW2 generations to be tested. This study expands on the previous analysis of age and cohort effects of the NCDS and BCS70 (Ploubidis, Sullivan, Brown, & Goodman, 2017; Sacker & Wiggins, 2002), by including additional waves of data (age 50 in NCDS and ages 34 and 46 in BCS70). In addition, the age profile of psychological distress is studied for the first time in the NSHD, the longest continuous follow-up of this outcome within the same individuals from age 36 to 69.

We hypothesised that (1) psychological distress increased between early-adulthood and midlife across all three birth cohorts; which had been preceded by elevated distress in the 20s and (2) psychological distress declined from midlife into early older age in the NSHD.

## Method

### Study population and design

We excluded those who died, emigrated from Britain or did not have at least one valid measure of psychological distress between age 23 and 69, which resulted in the analytical longitudinal sample: *n* = 3093 for NSHD, *n* = 13 250 for NCDS and *n* = 12 019 for BCS70 (online Supplementary eFig. 1). The prevalence of psychological distress is also provided using cross-sectional samples attained at each data sweep, also excluding those who died or emigrated (age 23–26: NCDS and BCS70; age 30: BCS70; age 33–36: all cohorts; age 42–43: all cohorts; age 50–53: NSHD and NCDS).

### Measures

Except for the Present State Examination (see below), the measures used in this study were not designed to diagnose any specific mental health condition, but rather capture general psychopathology (psychological distress) of common mental disorders, such as depression and anxiety. Both continuous and discrete versions of the outcomes were used. We derived a binary indicator of caseness, based on validated thresholds (see Table 1), aiming to identify individuals with scores high enough to represent a potential clinical diagnosis of common mental disorder.

**Table 1.** Details of measures of mental health used across the cohorts

Measure	Details	Caseness threshold	Psychometric (reliability/validity) and clinical properties
Present State Examination	A clinical examination, conducted by a nurse, assessing the frequency and severity of a range of psychiatric symptoms in the preceding month. Used at age 36 in NSHD.	5 or higher on the Index of Definition (Wing et al., 1974)	Tested in the general population: high agreement with the clinical diagnosis of common mental health disorders (≈90%); high concurrent validity with other measures of psychological distress (Wing et al., 1974). A diagnostic tool, hence information on specificity and sensitivity not provided.
Psychiatric Symptoms Frequency	18 items (based on the Present State Examination); 5-point scale (0 = never in the last year; 1 = up to 10 days in total, less than once a month; 5 = every day in the last year); assessing on symptoms of anxiety and depression during the preceding year; administered by a nurse. Used at age 43 in NSHD.	23 or higher on the summed up score (Lindelow et al., 1997)	Tested in the general health care/general population (age ≈ 43); Cronbach's alpha = 0.88; all items identified a common factor; AUC^a^ against reports of contact with a doctor/use of prescribed medication for ‘nervous or emotional trouble or depression’ (0.84–0.86) (Lindelow et al., 1997).
GHQ-28	28 items assessing symptoms of anxiety and depression in the preceding 4 weeks; a 1–4 point Likert scale and recoded into binary values; self-administered. Used at ages 50–69 at NSHD.	5 or higher endorsed symptoms (Goldberg et al., 1997)	Tested in the general health care (adults): Cronbach's alpha = 0.82–0.86 (Goldberg et al., 1997); all items identified 4 factors explaining 62% variance in psychological distress (Goldberg & Hillier, 1979); AUC^a^ against diagnosed psychiatric morbidity (0.93) (Goldberg et al., 1997).
Malaise Inventory	A shorter 9-item version (as opposed to the full 24 items version); binary (‘yes–no’) response scale; self-administered. Used across all ages in BCS70 and NCDS.	4 or higher on the summed up score	Tested in the general population (age 23–33); Cronbach's alpha = 0.70–0.80; all items identify a common factor; AUC^a^ against self-reported diagnosed psychiatric morbidity = 0.77–0.79) (Rodgers, Pickles, Power, Collishaw, and Maughan, 1999).

a Sensitivity (the proportion of cases who are correctly identified) and specificity (the proportion of non-cases correctly identified) expressed as area under the curve (AUC) (Florkowski, 2008).

The NSHD included a range of measures of psychological distress: a clinical interview for the frequency and severity of psychiatric symptoms in the preceding month at age 36 (the Present State Examination; PSE) (Wing, Cooper, & Sartorius, 1974); an interviewer-administered 18-item instrument derived from the PSE, focusing on symptoms of anxiety and depression during the preceding year at age 43 (the Psychiatric Symptom Frequency; PSF) (Lindelow, Hardy, & Rodgers, 1997) and a self-administered questionnaire assessing symptoms of anxiety and depression in the preceding four weeks at ages 53, 60–64 and 69 (the 28-item General Health Questionnaire; GHQ) (Goldberg & Hillier, 1979). All measures were found to be psychometrically robust (see Table 1 for more details). Due to differences in the measures used within NSHD, the continuous version of the outcome was based on seven harmonised items, which captured the same symptoms (details to follow).

In the NCDS and BCS70, at all ages cohort members completed the Malaise Inventory (Rutter, Tizard, & Whitmore, 1970), a measure of psychological distress level, or depression and anxiety symptoms. The Malaise Inventory has good psychometric properties (McGee, Williams, & Silva, 1986) and has been used both in the general population and high-risk groups (Furnham & Cheng, 2015). Scalar invariance of the measure was found within and across the NCDS and BCS70, as well as between genders (Ploubidis et al., 2017; Ploubidis, McElroy, & Moreira, 2019) (online Supplementary eTable 2). This implies that the symptoms captured by the items of the Malaise Inventory were interpreted equivalently by the participants, regardless of their age, cohort membership or measurement modes used at different ages.

### Harmonisation of the continuous outcome

The main aim of the study was to investigate the profile of psychological distress within the same individuals in each birth cohort. As the instruments used within NSHD differed, it was necessary for the study aims to select comparable items from each measure ensuring that their understanding is the same across age (resulting in a 7-item subset; see online Supplementary eAppendix 1 & eTable 1 for details). This was not an issue for NCDS and BCS70, since the same measure – the Malaise Inventory (Rutter et al., 1970) – was used for both and was found to be invariant across age and birth cohorts (see online Supplementary eTable 2). Nonetheless, to facilitate cross-cohort comparisons we also used a harmonised 4-item subset within and across all three birth cohorts. To the best of our knowledge, this is the first attempt to correct for measurement error due to different measures of mental health in the British birth cohorts.

Candidate items for harmonisation were identified by three independent raters – experienced psychologists and one active clinical practitioner. Initially, two raters scrutinised every available item within each measure administered in the cohorts and assigned each item a code reflecting its core content at the symptom level. In cases where the two raters disagreed, a third independent rater decided which item code (if either) was most appropriate (McElroy, Villadsen, Patalay et al., 2020). In this way, seven items were selected for the NSHD, which facilitated comparisons within this cohort. In addition, following the same process, four items were identified allowing for comparisons across the NSHD, NCDS and BCS70. Agreement between the raters was high (88%).

The harmonised items were summed and correlated with the full, original scale scores. For the 7-item subset, these correlations ranged from 0.81 (GHQ) to 0.85 (PSF), while for the 4-item subset they ranged from 0.78 (PSF) to 0.98 (Malaise). Crucially for the study aim, this demonstrated that the rank ordering of participants was closely comparable across subscales including harmonised items and the original full scales. To further test whether the two sets of harmonised items were comparable, we formally tested their measurement equivalence within NSHD (seven items) and across all cohorts (four items). Measurement equivalence (or invariance) was formally tested with latent variable multigroup models that allow researchers to verify the degree to which items function equivalently and therefore can be reliably compared across groups (Meredith, 1993; Ploubidis et al., 2019). In our case, ‘groups’ were defined by age within NSHD and by age and cohort across all three cohorts.

Scalar invariance was obtained for subscales consisting of the seven harmonised items within the NSHD (online Supplementary eTable 3). Thus, we can conclude that the 7-item subset is highly comparable across ages within NSHD. Concerning the 4-item subset, partial scalar invariance was observed (online Supplementary eTable 4), thus it can be used for comparisons of mean-level scores (i.e. summed-up total) within and across cohorts (Asparouhov & Muthen, 2014).

### Missing data

The extent of missing data increased in progressively younger cohorts (online Supplementary eTable 5); for instance, at age 42–43 the outcome data were missing in 12.2% of the eligible sample in NSHD, 20.8% in NCDS and 35.5% in BCS70 (online Supplementary eTable 5). The strategies to preserve sample representativeness and reduce bias were maximum likelihood in longitudinal models and multiple imputation with chained equations (20 imputations) for the cross-sectional prevalence rates (online Supplementary eTable 6), both under the missing-at-random (MAR) assumption (Collins, Schafer, & Kam, 2001). The MAR mechanism, which is largely untestable, implies that systematic differences between the missing values and the observed values can be explained by observed data (Collins et al., 2001), which has been found highly plausible in the British birth cohorts (Mostafa et al., 2020). The aim of multiple imputation was to provide robust cross-sectional estimates of psychological distress. The age, cohort and gender-stratified imputation models included measures of psychological distress, sampling weights and auxiliary variables (birthweight and parental socioeconomic status). In addition, each model included previous measures of psychological distress, which was a powerful predictor of both the outcome and missingness (online Supplementary eTable 5). We aimed to keep the models as consistent across cohorts as possible to ensure that potential differences in cross-cohort distributions were not due to varying approaches to data missingness.

### Age trajectories of psychological distress

We used the multilevel growth curve framework – with logit models for binary outcomes and Poisson models for continuous (count) outcomes. This framework allows for modelling data that are unbalanced in time and include missing data (Raudenbush & Bryk, 2002). It also accounts for a hierarchical dependency of observations (level 1) within individuals (level 2) – with age becoming an observation-level variable (Suzuki, 2012).

A similar modelling strategy was employed for both types of outcomes. Squared or cubic age polynomial terms were included and retained if their inclusion improved model fit [lower Akaike information criterion (AIC) and Bayesian information criterion (BIC)]. All-models were adjusted for gender. The fixed part of all models included age, gender and the intercept. The random part of the model captured variance in the intercept. Random age slopes were not included as the models resulted in non-positive definite matrices, possibly due to highly unbalanced data (Singer & Willet, 2003) and/or not enough variation around the age slope. All models included weights to account for the social class-stratified sample of the NSHD, with participants from the NCDS and BCS70 being given the weighting value of one. The cohort- and age-stratified estimates from the model, obtained with predictive margins, were compared to cross-sectional values that did not rely on the mathematical shape functions of the model.

In the analysis with the 4-items subset, age-by-cohort interaction was additionally tested in the fixed part of the model, at the significance level of *p* < 0.05 and according to AIC and BIC. This formally examined if the age profile of psychological distress was universal across the three birth cohorts. Finally, using comparable items across and within cohorts allowed for modelling the age profile of psychological distress pooled across all cohorts – resulting in a curve capturing growth based on observations from age 23 to 69. All analyses were conducted using STATA 15 (StataCorp, 2017).

### Supplementary analysis – age distribution of individual symptoms

We plotted the cohort-stratified age profile of each symptom from the harmonised 4-item subset to investigate whether all symptoms followed similar age distribution as the aggregated scales.

## Results

### Age distribution of psychological distress

The cross-sectional proportion of cases was highest in midlife in all three cohorts (i.e. 19.1% at age 53 in NSHD, 15.2% at age 50 in NCDS, 19.9% at age 46 in BCS70) (MI column in online Supplementary eTable 6).

### Profile of psychological distress across adulthood (age effects)

**Fig. 1.**
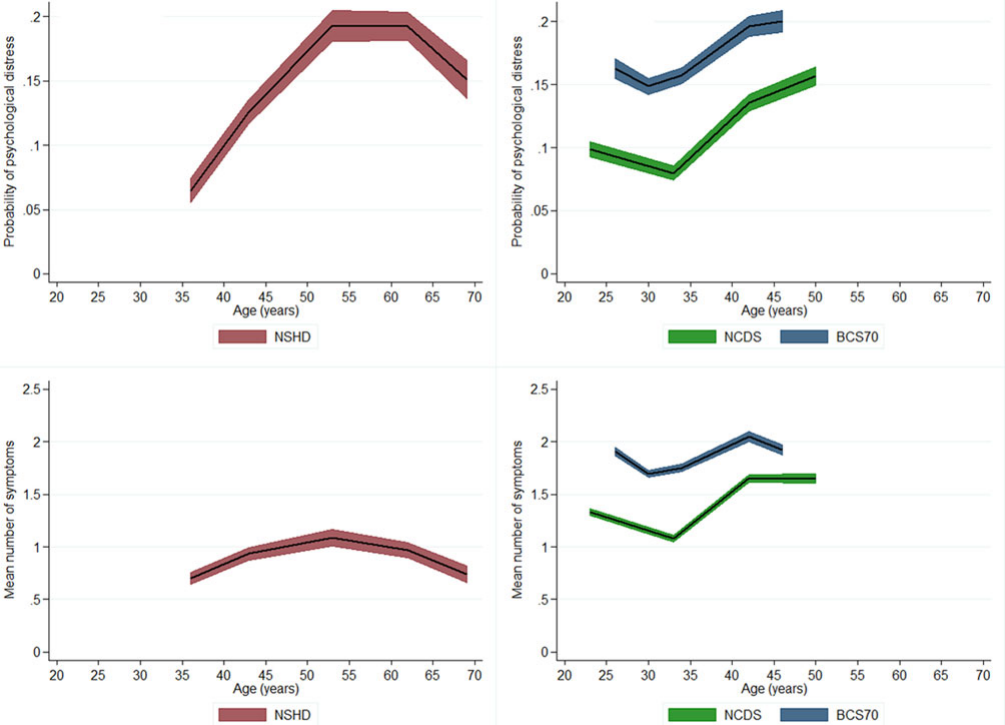
Cohort-stratified age profile of psychological distress – as a binary and continuous outcomes.

The age profile of psychological distress followed a quadratic shape in the NSHD and cubic in NCDS as well as BCS70 (see Fig. 1 – panels A and B and online Supplementary eTable 7 for details). Both in the NCDS and BCS70, there was an initial drop in the predicted probability of being a case, obtained from the multilevel logit model, between age 23–26 and 33–34. Subsequently, there was an increase in the probability of caseness between early-adulthood and midlife in all three cohorts. Probability of being a case increased from 12.5% at age 36 to 19.5% at age 50–53 in NSHD; from 8.0% at age 33 to 13.7 at age 42 in NCDS and from 15.7% at age 34 to 19.7% at age 42 in BCS70 (MLR column in online Supplementary eTable 6). In the NSHD, where the data were collected until early old age, there was a drop in the probability of caseness between age 60–64 and 69 (19.5% *v.* 15.2%).

The age profile of continuous outcomes was highly comparable to the one obtained with the binary outcome, also having a quadratic shape in the NSHD and cubic in both the NCDS and BCS70 (see Fig. 1 – panels C and D and online Supplementary eTable 8 for details). However, there were slight differences in the trajectories across both types of outcomes (see online Supplementary eAppendix 2 and eFig. 2 for more details). Overall, women had higher levels of psychological distress across all birth cohorts when either version of the outcome was used (online Supplementary eTables 7 and 8; eFigs 3 and 4).

### Age and cohort effects on psychological distress – based on the 4-items subset

**Fig. 2.**
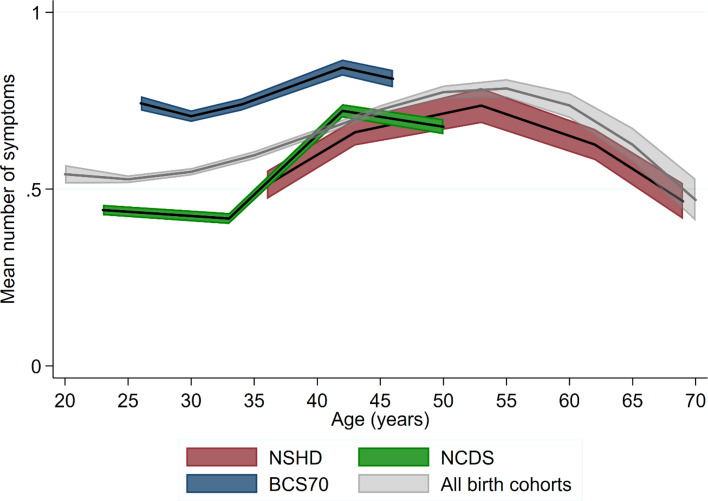
Age profile of the mean number of symptoms – cohort-stratified and pooled across cohorts.

Harmonisation of individual items allowed for direct comparisons across all three cohorts as well as for modelling the age profile based on observations from age 23 to 69 – after pooling all measures across the cohorts. Overall, the age trajectories within all the cohorts were comparable to those obtained in the analysis using binary and other continuous versions of the outcome (Fig. 2). We further tested if the increase from early-adulthood (age 33–36) to midlife (42–43) was uniform in relative terms across the cohorts. There was evidence for a steeper increase in symptoms in NCDS [*B* = 0.51, 95% confidence interval (CI) 0.48–0.53] and BCS70 (*B* = 0.48, 95% CI 0.45–0.50) compared with NSHD (*B* = 0.38, 95% CI 0.30–0.46); no difference was found between NCDS and BCS70. Overall, the BCS70 had worse psychological distress than two other cohorts across at overlapping age (26–46) (Fig. 2).

**Fig. 3.**
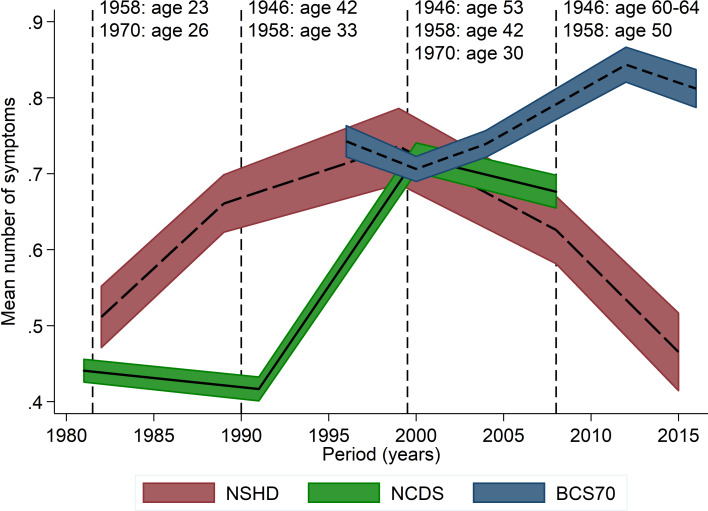
Cohort stratified age profile of the mean number of symptoms – with calendar years on the *X*-axis.

Considering all birth cohorts simultaneously also allowed for exploring potential period effects by plotting trajectories with calendar years instead of age on the *X*-axis (Fig. 3). The increase in psychological distress from early-adulthood to midlife occurred during the 1990s in the NSHD and NCDS, which may suggest at least a partial influence of period effects. However, a similar age profile at the same life-phase was also observed for the BCS70 from the year 2000 onwards, in which psychological distress started to decline in two other cohorts. This is consistent with the increase between early-life and midlife being mostly attributed to an age effect.

### Age distribution of individual symptoms – exploratory analysis

**Fig. 4.**
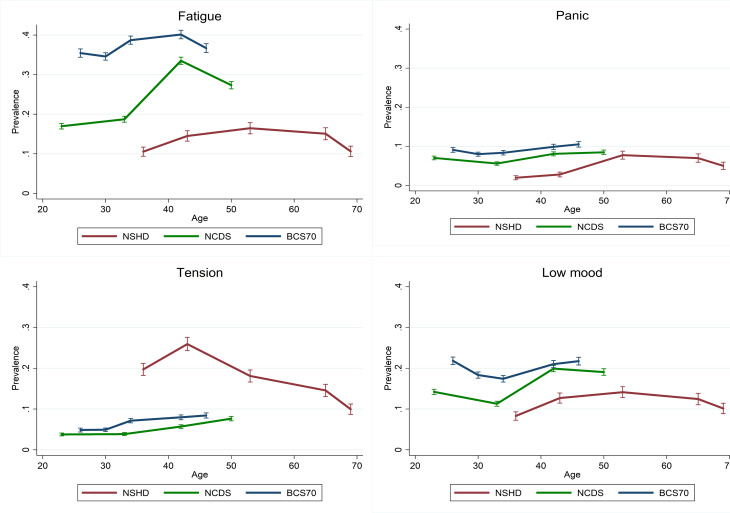
Cohort-stratified age distribution of individual symptoms.

Fatigue and low mood were the most prevalent symptoms within the harmonised 4-item subset; these followed a very similar age profile as the main outcome measures in all three cohorts (Fig. 4). The age curve of the panic symptom, however, was much flatter in the NCDS and BCS70 (Fig. 4). Finally, tension appeared to marginally increase during the entire adulthood in the NCDS and BCS70, whereas it peaked quite visibly at age 43 in the NSHD and subsequently declined (Fig. 4).

## Discussion

### Main findings

Across three post-war British birth cohorts, there was a consistent increase in psychological distress from early adulthood to midlife (between ages 30–36 and 42–43). This finding was largely unaffected by differences in outcome definitions (continuous *v.* binary), missing data strategy (FIML *v.* multiple imputation) and sample definition (cross-sectional *v.* longitudinal). The increase in midlife appears to be steeper in the NCDS and BCS70 than in the NSHD. In the NSHD, where additional data sweeps were available, psychological distress subsequently declined between midlife and early old age. Participants of both NCDS and BCS70 also experienced elevated levels of psychological distress in their mid-20s compared with their early-mid 30s. Overall, after controlling for cohort differences, the age profile of psychological distress followed an inverted U-shape in adulthood, with symptoms increasing from early- to mid-adulthood, and subsequently declining. Furthermore, the BCS70 reported more psychological distress compared to the two other cohorts across at overlapping age (26–46).

### Comparison with other studies

The most common pattern of the age distribution of psychological distress in the literature is an initial rise in psychological distress until around age 45–55, followed by a drop into older age (Jorm, 2000). The most consistent finding is that psychological distress and other related mental health outcomes worsen between early-adulthood and midlife (Bell, 2014; Blanchflower & Oswald, 2008; Jokela et al., 2013; Sacker & Wiggins, 2002). However, in our study, consistently with previous findings using these data (Sacker & Wiggins, 2002), members of both the NCDS and BCS70 experienced higher psychological distress in mid-20s than in early-30s. Elevated levels of psychological distress across adulthood were also found among 20–24 year-olds in Canada and the USA born in 1970s–1980s, in repeated cross-sectional studies conducted between 1997 and 2008 (Keyes et al., 2014).

The finding of elevated psychological distress in the mid-20s has been rarely discussed in the literature in the UK. This may be due to modelling strategies masking this effect, such as fitting polynomial age trajectories and pooling data across several ‘pseudo cohorts’ to obtain a profile with a longer age range. For instance, worse mental health can be observed in the British Household Panel Survey between ages 15–24 and 25–34 in cohorts born between 1960s and 1980s, when age was treated as a categorical variable (Jokela et al., 2013). However, when age trajectories within the cohorts were modelled as cubic polynomials, the apparent higher distress in the early-20s was not detected (Bell, 2014). A similar observation can be made in the British Adult Psychiatric Morbidity Survey, where mental health problems increased in the mid-20s and subsequently declined in the cohorts born in 1964–1970 (among women only) and 1971–1977 (Spiers et al., 2012). Nonetheless, when the age profile was pooled across different cohorts, it had an inverted U-shape (Spiers et al., 2012).

This can also be observed in our study – both in descriptive cross-sectional data and when age was modelled across individual birth cohorts, where we saw elevated psychological distress in mid-20s in NCDS and BCS70. However, modelling age profiles across all three birth cohorts resulted in the curve having an inverted U-shape – with a flattening tail in mid-20s where predicted values were slightly underestimated compared to the observed data.

Further research is needed to understand the extent to which the elevated psychological distress is a cohort effect – applying mostly to those born in 1960s and 1970s and to what extent this is an age effect experienced also across younger birth cohorts. In the UK, this research question can be tested with the Next Steps study (the cohort born in 1989–90), the Avon Longitudinal Study of Parents and Children (born in 1991–1992), and in the near future, the Millennium Cohort Study (born 2000–2001).

Another inconsistency in the evidence is the apparent improvement in mental health between midlife and early older age. Studies based on the British Household Panel Survey (1991–2008) found a peak in distress around age 45, followed by a decline and a subsequent rise at old age (Bell, 2014; Jokela et al., 2013). However, the results were somewhat inconsistent depending on the methodology used. When age was modelled as a cubic-shaped curve, mental health appeared to worsen throughout adulthood, with a slow decline in midlife (Bell, 2014). Whereas, entering age as a categorical variable to the model, which did not impose any specific shape on the age profile, resulted in a clear drop in mental health problems between ages 45–54 and 65–74 (Jokela et al., 2013). Noteworthily, mental health started to deteriorate again after age 70 in both studies (Bell, 2014; Jokela et al., 2013). Similarly, a study based on the English Longitudinal Study of Ageing showed declining rates of mental health problems between ages 50 and 65, and subsequently increasing rates between ages 65 and 90 (Tampubolon & Maharani, 2017). Hence, it is likely that the NSHD may also experience worsening psychological distress in the subsequent waves of data collection, which future research can investigate.

### Interpretation and implications

This study helps to clarify age patterns in adulthood psychological distress using the longitudinal data collected over a long period and across various birth cohorts. This study design enabled us to investigate both age and cohort effects simultaneously. Establishing the association between age and psychological distress, while considering confounding effects of birth year, and potential biases due to measurement error and missing data, is an important step for evidence-based policy development. This may be particularly important for midlife individuals, who are at increased risk of psychological distress, particularly in younger birth cohorts. In this group, psychological distress may be partially responsible for the increases in premature mortality, for instance, due to its association with the cardiometabolic system (Winning, Glymour, McCormick, Gilsanz, & Kubzansky, 2015) or deaths of despair (Joyce & Xu, 2019), potentially leading to the recent stagnation of life expectancy in the UK (Leon, Jdanov, & Shkolnikov, 2019).

Overall, there is little theory to draw on when explaining age profiles of psychological distress in adulthood (Blanchflower & Oswald, 2008). Here, we discuss factors potentially linked with mental health changes at different phases of adulthood. Starting with the increase in poor mental health in mid-20s, it is most likely an age effect, as it was observed across different socioeconomic and historical contexts (at least across high-income countries). It was found across different periods: in 1981 (in NCDS) and 1996 (in BCS70) in our study, as well as between 2000 and 2007 in the study conducted by Spiers et al. (2012). In addition, this increase was also found across different birth cohorts ranging from the late-1950s to 1980s (Bell, 2014; Jokela et al., 2013; Spiers et al., 2012), and in different countries including the UK, Canada and the USA (Keyes et al., 2014). It has been argued that the age between 18 and 29 should be acknowledged as an important developmental period with major role shifts, as people leave their parental home and start developing their own home and family (Arnett, Zukauskiene, & Sugimura, 2014; Gustavson et al., 2018). Cohort effects, however, still may play a role contributing to higher distress among those born in 1970. This birth cohort was likely to experience instability in tenancy, and was particularly disadvantaged in their transition from education to work, as they entered the labour market in mid-1980s during high unemployment among young people (Sullivan et al., 2015). This may have had lasting effects on the psychological distress of this cohort throughout adulthood.

Processes underlying the observed increase in psychological distress from early-30s to midlife are unclear. Midlife tends to involve a ‘peak’ in career, with middle-aged adults acquiring increasing responsibility as the ‘decision-makers’ in society, which is accompanied by reduced leisure time (Willis, Martin, & Rocke, 2010). This is, for instance, reflected by elevated job-related stress in midlife (Health & Safety Executive, 2017) and it may provide a partial explanation for rising fatigue as observed in this study. Midlife individuals were found to experience declining quality and quantity of leisure time, as well as time with friends and family, which may translate into worse mental health (Otterbach, Sousa-Poza, & Møller, 2018). Middle-age is also often associated with changes to family structure, which may be linked with mental health, such as rising rates of divorce (Britton, Ben-Shlomo, Benzeval, Kuh, & Bell, 2015; Office for National Statistics, 2015b). At this life phase, people are more likely to be parents and simultaneously care for ageing parents (Office for National Statistics et al., 2016)

The reasons for the decline in psychological distress after midlife are also speculative. Selective mortality is one of the candidate explanations, as those in poorer mental health are at a greater risk of dying prematurely (Archer, Kuh, Hotopf, Stafford, & Richards, 2018). However, assuming that the mortality rates in the three cohorts are representative of those observed in their target populations (Wadsworth et al., 1992) and that absolute mortality rates have declined during the investigated period (Office for National Statistics, 2015a) – any effects of selective mortality due to mental health reflect a population selection process and are not sample-specific bias. Older individuals, particularly those in more advantaged social classes, may also experience improved mental health due to relief from major midlife stressors, for instance, due to retirement or stabilisation in family life. Indeed, the perceived daily stress reduces in this life phase; however, this reduction was not found to be associated with whether one was in full-time employment or with marital status (Stone, Schneider, & Broderick, 2017). It has also been suggested that older people shift from attainment-related goals, such as status or skills, towards those that help them maintain emotional stability – a phenomenon known as socioemotional selectivity (Carstensen, Fung, & Charles, 2003). For instance, older individuals may be more likely to invest in relationships and activities that are positive and rewarding whilst ceasing those that are not. This, in turn, helps them to confront stressors and adversity (Carstensen et al., 2003). It is also possible that mental health problems more specific to old age are not well-captured by conventional symptom scales, hence underestimating the frequency of distress (Mezuk & Kendler, 2012). For instance, physical symptoms of distress, such as decreased energy, fatigue or difficulty with sleeping, may be normalised and explained through deteriorating health related to ageing rather than emotional state (Christensen et al., 1999).

In the UK, the Royal College of Psychiatrists position statement ‘No health without public mental health’ (PS4/2010) recommends early intervention in the life course, since the majority of mental illnesses have childhood antecedents; and also recommends targeted approaches to promote mental health and prevent mental disorder in old age. However, there is no key recommendation for midlife mental ill health, a gap also reflected in the Royal College of General Practitioners Mental Health Toolkit. To some extent, this gap is addressed by Public Health England, but the emphasis is on mental health in the workplace (Mental Health Taskforce, 2016). Studies elsewhere highlight that women in midlife with psychological distress have significant unmet health care needs (Johnson, Jou, & Upchurch, 2020; Outram, Murphy, & Cockburn, 2004). Our study suggests that increased attention should be paid to the detection and management of psychological distress in midlife, for instance in primary care. It also implies the need for increased public awareness of mental health in midlife.

### Strengths and limitations

The main strength of this study is that it used three population-based prospective studies, including – to the best of our knowledge – the longest continuous follow-up of psychological distress within the same individuals, from ages 36 to 69. This is in contrast to most of the previous research that relied on statistical methods pooling observations from longitudinal or repeated cross-sectional surveys across multiple ‘pseudo’ cohorts. Another strength is that the same measure of distress was used in two of the cohorts, which was found to be invariant longitudinally, across the cohorts and genders (Ploubidis et al., 2019).

A key limitation is that different measures of psychological distress were used within the NSHD – this may specifically impair comparability at ages 36–53 – and between this cohort and the NCDS and BCS70. However, comparable items at the symptom level within and across cohorts were identified through a comprehensive and robust harmonisation process. This resulted in the harmonised subsets of items, which were further tested for measurement equivalence and were found to be invariant within NSHD (7-items) and across the three cohorts (4-items). Hence, their means can be compared within and across cohorts without bias (Asparouhov & Muthen, 2014). In addition, differences in measures are unlikely to solely explain our major finding – an increase in psychological distress from early-adulthood to midlife, followed by a decline in early old age. In the NCDS and BCS70, the same measure was used between ages 23 and 50 – the Malaise Inventory. Hence inferences regarding the age distribution of psychological distress within and across the NCDS *v.* BCS70 are robust. In the NSHD, we observed an increasing trend in psychological distress between ages 36 and 43, which is based on two closely-related measures increasing our confidence in findings. The Psychiatric Symptoms Frequency used at age 43 was developed based on the Present State Examination that was used at age 36 (Lindelow et al., 1997). Similarly, the observed decline in psychological distress between ages 53 and 69 in the NSHD cannot be attributed to differences in measures since the GHQ-28 was used at all three ages.

Our findings do not provide an estimate of the prevalence of common mental disorders or use of mental health services. Instead, they focus on a relative risk of depressive and anxiety symptoms across age. Furthermore, as psychological distress is limited to symptoms of depression and anxiety, it does not capture a range of other mental health problems in the population – such as psychosis or bipolar disorder.

Another limitation of our study, as with most longitudinal research, is a considerable amount of missing data. Our analysis relied on the MAR assumption that is not empirically verifiable (Little & Rubin, 2002). However, we increased the plausibility of the MAR assumption by including rich information on health and related variables available from birth in the imputation model (Mostafa & Wiggins, 2015). The information contributed by these auxiliary variables allows for predicting missing data with greater accuracy and minimising non-random variation in these values (Sterne et al., 2009). Besides, obtaining consistent findings when using different missing data strategies – multiple imputation and full information maximum likelihood (Enders, 2010) – further increased the robustness of our analyses.

## Conclusion

Across three post-war British cohorts, there was a consistent increase in psychological distress from early-adulthood to midlife (between ages 30–36 and 42–43). In the NSHD, where additional data sweeps were available, psychological distress diminished into early old age. There is a need for further research to understand processes underlying elevated psychological distress at each life phase (early and mid-20s as well as 40s–50s) and for cross-cohort differences. The British birth cohorts, including those following younger participants, are well-suited for studying those mechanisms.

## Supplementary material

For supplementary material accompanying this paper visit https://doi.org/10.1017/S003329172000327X. click here to view supplementary material
