# Supplementary material for: Psychological distress from early adulthood to early old age: evidence from the 1946, 1958 and 1970 British birth cohorts
Source: Psychol Med. 2021 Jan 21;52(8):1471–80. doi: 10.1017/S003329172000327X (PMC9226427; doi:10.1017/S003329172000327X)
Supplement: Supplementary file 1 [file S003329172000327Xsup001.docx]

eAppendix 1. Harmonisation of the comparable items across the birth cohorts.

eTable 1. Harmonised items across measures of psychological distress.

eTable 2. Longitudinal examination of the measurement equivalence of the Malaise Inventory in the NCDS and BCS70 (adapted from Ploubidis, McElroy & Moreira, 2019).

eTable 3. Longitudinal examination of the measurement equivalence of seven harmonised items in the NSHD.

eTable 4. Longitudinal examination of the measurement equivalence of four harmonised items in the NSHD, NCDS, and BCS70.

eTable 5. Frequency and predictors of missing data in the outcome.

eTable 6. Age distribution of caseness (as a binary outcome) based on multiple imputation and multilevel logit regression.

eTable 7. Age profile of psychological distress in the NSHD, NCDS, and BCS70—estimates from multilevel logit regression based on the binary outcome.

eTable 8. Age profile of psychological distress in the NSHD, NCDS, and BCS70—estimates from multilevel Poisson regression based on the continuous outcome.

eAppendix 2. Age distribution of participants with varying proportions of symptoms.

eFigure 2. Cohort-stratified age distribution of participants with varying proportions of symptoms.

eFigure 3. Gender-stratified age profile of psychological distress in the NSHD, NCDS, and BCS70—estimates from multilevel logit regression based on the binary outcome.

eFigure 4. Gender-stratified age profile of psychological distress in the NSHD, NCDS, and BCS70—estimates from multilevel Poisson regression based on the continuous outcome.

eFigure 1. Flow diagram of the study sample.


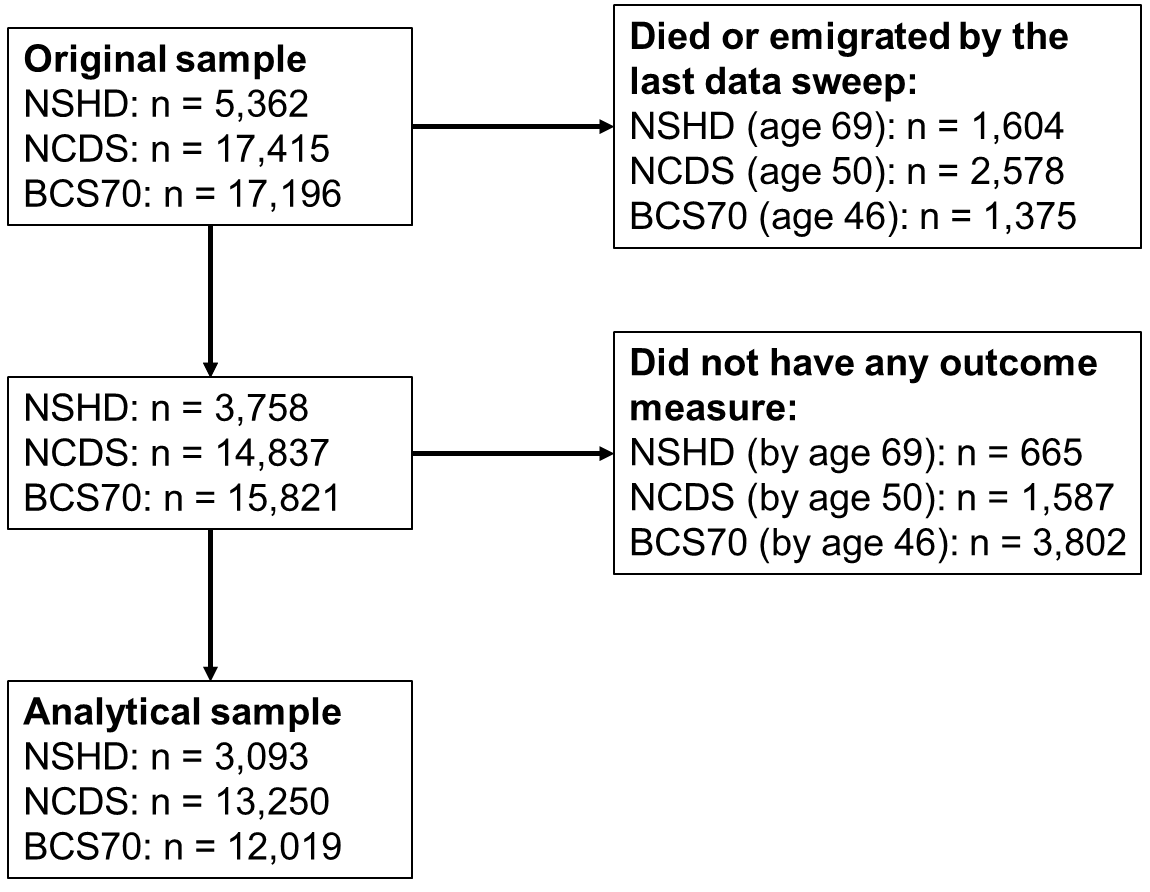


**eAppendix 1. Harmonisation of comparable items across the birth cohorts.**

It was necessary to synchronise the response scales across the measures. As transforming the binary response of the Malaise was not possible, the other three measures were recoded to a binary format. Every item from the GHQ, PSF, and PSE was recoded as either 0 (absence of symptom) or 1 (endorsement of symptom). The (0-0-1-1) scoring was used for the GHQ, with “worse/more than usual” or “much worse/more than usual” corresponding to an endorsement of a symptom. Symptoms within the PSE were considered as endorsed if present regardless of clinical severity. Finally, within the PSF endorsement of symptoms was indicated by responding “quite often”, “often” or “always” to a question.

| eTable 1. Harmonised items across measures of psychological distress. | | | | |
| --- | --- | --- | --- | --- |
| Symptom | **Present State Examination** | **Psychiatric Symptom Frequency Questionnaire** | **General Health Questionnaire** | **Malaise Inventory** |
| Low Mood | Do you keep reasonably cheerful or have you been very depressed or low spirited recently? (rate depressed mood) | Over the last year have you been in low spirits or felt miserable? | Have you recently been able to enjoy your normal day-to-day activities? | Do you often feel miserable or depressed? |
| Fatigue | Have you been exhausted and worn out during the day or evening even when you haven’t been working very hard? (rate tiredness/exhaustion) | Over the last year have there been days when you tired out very easily? | Have you recently been feeling in need of a good tonic? | Do you feel tired most of the time? |
| Tension | Do you often feel on edge, keyed up, mentally tense or strained? (rate nervous tension) | Over the last year have you felt on edge, keyed up or mentally tense? | Have you recently felt constantly under strain? | Are you constantly keyed up and jittery? |
| Panic | Have you had times when you felt shaky or you heart pounded or you felt sweaty and you simply had to do something about it? | Over the last year have you been in situations when you felt shaky or sweaty or your heart pounded or you could not get your breath? | Have you recently been getting scared or panicky for no good reason? | Does your heart often race like mad? |
| Hopelessness* | How do you see the future? (rate hopelessness) | Over the last year have you had the feeling that the future does not hold much for you? | Have you recently felt that life is entirely hopeless? |  |
| Health anxiety* | Do you tend to worry over your physical health? (rate hypochondriasis) | Over the last year have you been frightened or worried about becoming ill or about dying? | Have you recently felt that you are ill? |  |
| Sleep problems* | Have you had any trouble getting off to sleep in the last month?  (rate delayed sleep) | Over the last year have you had trouble getting off to sleep? | Have you recently lost much sleep over worry? |  |
| Response options | Symptom not present/ Symptom definitely present during past month, but of moderate clinical intensity/ Intense form of symptom present for more than 50% of past month | Never/ Occasionally/ Sometimes/ Quite often/ Very often/ Always | Not at all been feeling in need of a good tonic?)/ No more than usual/ Rather more than usual/ Much more than usual | No/Yes |
| *Additional items used for investigating age effects in psychological distress within the 1946 cohort (NSHD). | | | | |

eTable 2. Longitudinal examination of the measurement equivalence of the Malaise Inventory in the NCDS and BCS70 (adapted from Ploubidis, McElroy & Moreira, 2019).

|  |  |  | **Chi-square (d.f.)** | **RMSEA** | **CFI** | **TLI** | **ΔRMSEA** | **ΔCFI** | **ΔTLI** |
| --- | --- | --- | --- | --- | --- | --- | --- | --- | --- |
|  | ***NCDS 23, 33, 42, 50** | Configural | 1500.343 (216) | 0.033 (0.031 to 0.034 | 0.988 | 0.984 |  |  |  |
|  |  | Scalar | 2482.153 (265) | 0.039 (0.037 to 0.040) | 0.979 | 0.977 | 0.006 | 0.009 | 0.007 |
|  | ****BCS70 26, 30, 34, 42, 46** | Configural | 2169.417 (270) | 0.039 (0.038 to 0.041) | 0.986 | 0.982 |  |  |  |
|  |  | Scalar | 2815.072 (333) | 0.040 (0.039 to 0.042) | 0.982 | 0.981 | 0.001 | 0.004 | 0.001 |
|  | *****NCDS & BCS70 23/26, 33/34, 42** | Configural | 2354.059 (324) | 0.035 (0.033 to 0.036) | 0.986 | 0.981 |  |  |  |
|  |  | Scalar | 3774.957 (401) | 0.040 (0.039 to 0.041) | 0.976 | 0.974 | 0.005 | 0.010 | 0.007 |

*****Eight independent groups multigroup models (4 waves, gender)

******Ten independent groups multigroup models (5 waves, gender)

*** Twelve independent groups multigroup models (3 waves, 2 cohorts, gender)

| eTable 3. Longitudinal examination of the measurement equivalence of seven harmonised items in the NSHD (adapted from McElroy, Villadsen, Patalay et al., 2020). | | | | | | | | |
| --- | --- | --- | --- | --- | --- | --- | --- | --- |
| **Model** | **N** | **Chi-square (DF)** | **RMSEA** | **CFI** | **TLI** | **ΔRMSEA** | **ΔCFI** | **ΔTLI** |
| Configural | 13,886 | 544.328 (70) | 0.049 | 0.979 | 0.968 |  |  |  |
| Scalar* |  | 1175.735 (94) | 0.064 | 0.952 | 0.946 | 0.015 | 0.027 | 0.02 |
| Partial Scalar*^¥^ |  | 1173.975 (95) | 0.064 | 0.952 | 0.947 |  |  |  |
|  |  |  |  |  |  |  |  |  |
| *Latent variances fixed to 1  ^¥^Threshold for ‘tense’ freed | | | | | | | | |

| eTable 4. Longitudinal examination of the measurement equivalence of four harmonised items in the NSHD, NCDS, and BCS70 (adapted from McElroy, Villadsen, Patalay et al., 2020). | | | | | | | | |
| --- | --- | --- | --- | --- | --- | --- | --- | --- |
| **Model** | **N** | **Chi-square (DF)** | **RMSEA** | **CFI** | **TLI** | **ΔRMSEA** | **ΔCFI** | **ΔTLI** |
| Configural | 65,997 | 269.102 (18) | 0.044 | 0.994 | 0.983 |  |  |  |
| Scalar |  | 4087.048 (66) | 0.091 | 0.914 | 0.929 | 0.047 | 0.08 | 0.054 |
| Partial Scalar* |  | 1129.715 (58) | 0.050 | 0.976 | 0.977 | 0.006 | 0.018 | 0.006 |
| Partial Scalar 2** |  | 444.496 (50) | 0.033 | 0.991 | 0.990 | 0.011 | 0.003 | 0.007 |
| *Threshold for ‘tense’ freed  **Thresholds for ‘tense’ and ‘fatigue’ freed | | | | | | | | |

| eTable 5. Frequency and predictors of missing data in the outcome. | | | | | | |
| --- | --- | --- | --- | --- | --- | --- |
| **N (eligible sample)** | **Age 33-34** | | **Age 42** | | **Age 50-53** | |
|  | n (%) | | n (%) | | n (%) | |
| 1946 (n=3,093) | 376 (12.16) | | 376 (12.16) | | 520 (16.81) | |
| 1958 (n=13,250) | 2,854 (21.54) | | 2,751 (20.76) | | 4,129 (31.16) | |
| 1970 (n=12,019) | 3,287 (27.35) | | 4,270 (35.53) | |  | |
|  | Missing outcome | Outcome | Missing outcome | Outcome | Missing outcome | Outcome |
| Logistic regression estimates | RR (95%CI) | RR (95%CI) | RR (95%CI) | RR (95%CI) | RR (95%CI) | RR (95%CI) |
| Birth cohort (1946 – reference category) |  | |  | |  | |
| 1958 | 1.76 (1.64, 1.88) | 1.32 (1.17, 1.49) | 1.61 (1.50, 1.72) | 1.06 (0.97, 1.15) | 1.77 (1.67, 1.87) | 0.77 (0.72, 0.83) |
| 1970 | 2.23 (2.08, 2.39) | 2.70 (2.41, 3.02) | 2.75 (2.58, 2.94) | 1.48 (1.37, 1.61) |  | |
|  |  | |  | |  | |
| Women (men – reference category) | 0.83 (0.80, 0.87) | 1.82 (1.68, 1.97) | 0.85 (0.82, 0.89) | 1.53 (1.44, 1.63) | 0.96 (0.94, 0.98) | 1.62 (1.51, 1.75) |
|  |  | | | |  | |
| Non-manual father’s occupational class  (manual – reference) | 1.23 (1.16, 1.30) | 1.24 (1.14, 1.36) | 1.21 (1.16, 1.27) | 1.28 (1.19, 1.38) | 1.27 (1.19, 0.96) | 1.07 (0.98, 1.16) |
|  |  | |  | |  | |
| Psychological distress at age 15/16 | 1.17 (1.07, 1.28) | 2.19 (1.95, 2.46) | 1.15 (1.06, 1.25) | 2.05 (1.86, 2.25) | 1.04 (1.00, 1.09) | 1.60 (1.42, 1.80) |
|  |  | |  | |  | |
| Psychological distress at preceding age | 1.28 (1.19, 1.38) | 6.25 (5.72, 6.84) | 1.62 (1.50, 1.74) | 5.32 (5.00, 5.67) | 1.26 (1.22, 1.31) | 3.85 (3.58, 4.13) |
|  |  | |  | |  | |
| Normal birthweight (low: <2500g – reference) | 0.91 (0.83, 0.99) | 0.80 (0.68, 0.93) | 0.87 (0.81, 0.95) | 0.85 (0.74, 0.97) | 0.91 (0.87, 0.95) | 0.82 (0.70, 0.96) |

| eTable 6. Age distribution of caseness (as a binary outcome) based on multiple imputation and multilevel logit regression. | | | | | | | | |
| --- | --- | --- | --- | --- | --- | --- | --- | --- |
|  | **NSHD** | |  | **NCDS** | |  | **BCS70** | |
|  | **MI** | **MLR** |  | **MI** | **MLR** |  | **MI** | **MLR** |
|  | **%** (95%CI) | **%** (95%CI) |  | **%** (95%CI) | **%** (95%CI) |  | **%** (95%CI) | **%** (95%CI) |
| Age 23-26 |  |  |  | 9.6 (9.0, 10.1) | 9.9 (9.3, 10.5) |  | 16.0 (15.2, 16.8) | 16.3 (15.5, 17.1) |
| Age 30 |  |  |  |  | |  | 13.7 (13.1, 14.4) | 14.8 (14.2, 15.4) |
| Age 33-36 | 6.0 (5.1, 6.9) | 6.2 (5.4, 7.2) |  | 7.9 (7.3, 8.4) | 8.0 (7.5, 8.5) |  | 16.1 (15.4, 16.9) | 15.7 (15.1, 16.3) |
| Age 42-43 | 11.9 (10.7, 13.1) | 12.5 (11.6, 13.5) |  | 13.3 (12.6, 13.9) | 13.7 (13.0, 14.4) |  | 18.7 (17.9, 19.4) | 19.7 (18.9, 20.5) |
| Age 46 |  |  |  |  | |  | 19.9 (19.0, 20.1) | 20.0 (19.2, 20.1) |
| Age 50-53 | 19.1 (17.6, 20.7) | 19.5 (18.3, 20.7) |  | 15.2 (14.3, 16.0) | 15.8 (15.1, 16.6) |  |  | |
| Age 60-64 | 18.1 (16.5, 19.6) | 19.5 (18.4, 20.6) |  |  | |  |  | |
| Age 69 | 14.8 (13.3, 16.3) | 15.2 (13.7, 16.8) |  |  | |  |  | |
| MI = estimates based on multiple imputation; MLR = predicted probability from the multilevel logit regression. | | | | | | | | |

| eTable 7. Age profile of psychological distress in the NSHD, NCDS, and BCS70—estimates from multilevel logit regression based on the binary outcome. | | | | | |
| --- | --- | --- | --- | --- | --- |
|  | **NSHD** |  | **NCDS** |  | **BCS70** |
|  | Coef. (95%CI) |  | Coef. (95%CI) |  | Coef. (95%CI) |
| Intercept | -20.25 ( -22.86, -17.65) |  | 13.76 (9.99, 17.53) |  | 14.43 (7.50, 21.36) |
| Intercept variance | 7.43 (6.43, 8.59) |  | 5.60 (5.06, 6.20) |  | 5.70 (5.23, 6.21) |
| Age | 0.55 (0.45, 0.64) |  | -1.73 (-2.06, -1.39) |  | -1.60 (-2.20, -1.00) |
| Age^2^ | -0.00 (-0.01, -0.00) |  | 0.05 (0.04, 0.06) |  | 0.04 (0.03, 0.06) |
| Age^3^ |  |  | -0.00 (-0.00, -0.00) |  | -0.00 (-0.00, -0.00) |
| Woman (vs man) | 1.12 (0.91, 1.33) |  | 1.20 (1.07, 1.34) |  | 0.73 (0.61, 0.85) |
| Observations | 12,229 |  | 41,177 |  | 41,466 |
| Participants | 3,093 |  | 13,250 |  | 12,019 |
| AIC | 16176.28 |  | 24799.77 |  | 31684.08 |
| BIC | 16213.33 |  | 24851.53 |  | 31735.87 |
| *Note.* AIC = Akaike information criterion; BIC = Bayesian information criterion. | | | | | |

| eTable 8. Age profile of psychological distress in the NSHD, NCDS, and BCS70—estimates from multilevel Poisson regression based on the continuous outcome. | | | | | |
| --- | --- | --- | --- | --- | --- |
|  | **NSHD** |  | **NCDS** |  | **BCS70** |
|  | Coef. (95%CI) |  | Coef. (95%CI) |  | Coef. (95%CI) |
| Intercept | -5.28 (-6.16, -4.40) |  | 9.72 (8.90, 10.55) |  | 1.41 (1.09, 1.84) |
| Intercept variance | 2.17 (1.99, 2.37) |  | 0.97 (0.93, 1.00) |  | 0.78 (0.75, 0.81) |
| Age | 0.16 (0.13, 0.20) |  | -0.97 (-1.04, -0.90) |  | -0.96 (-1.08, -0.83) |
| Age^2^ | -0.00 (-0.00, -0.00) |  | 0.03 (0.03, 0.03) |  | 0.03 (0.02, 0.03) |
| Age^3^ |  |  | -0.00 (-0.00, -0.00) |  | -0.00 (-0.00, -0.00) |
| Woman (vs man) | 1.78 (1.59, 2.00) |  | 0.59 (0.55, 0.64) |  | 0.37 (0.33, 0.41) |
| Observations | 12,229 |  | 41,177 |  | 41,466 |
| Participants | 3,093 |  | 13,250 |  | 12,019 |
| AIC | 55055.44 |  | 119340.8 |  | 135142.5 |
| BIC | 55085.08 |  | 119392.6 |  | 135194.3 |
| *Note.* AIC = Akaike information criterion; BIC = Bayesian information criterion. | | | | | |

**eAppendix 2. Age distribution of participants with varying proportions of symptoms.**

The curve in the NSHD was slightly flatter, where the increase in psychological distress in mid-life was not as severe as the rise in the proportion of cases. In the NCDS, the predicted mean number of symptoms remained relatively stable between age 42 and 50 (1.65, 95%CI 1.61 to 1.69 vs 1.64, 95%CI 1.60 to 1.69), whereas the probability of being a case increased marginally in the same age range (13.7%, 95%CI 13.0 to 14.4 vs 15.8% 95%CI 15.1 to 16.6). In the BCS70, the mean number of symptoms declined between age 42 and 46 (2.04, 95%CI 1.99 to 2.09 vs 1.91, 95%CI 1.86 to 1.96) when the continuous outcome was used, which was in contrast to stable probability of caseness (19.7% 95%CI 18.9 to 20.5 vs 20.0% 95%CI 19.2 to 20.1).

Considering longitudinal changes in proportions of those with a different number of symptoms may help to understand the discrepancies between the binary and continuous outcomes. As presented by eFigure 2, it appears that the initial increase in psychological distress in early-40s was driven by declining proportion of those with no symptoms and increase in the proportion of those with any number of symptoms. Hence, in this life stage binary and continuous outcomes show similar results. Subsequently in mid-40s and early-50s, the proportions of those with no symptoms and with four or more symptoms (or three or more in NSHD) increased. Whereas individuals who had between one and three (or one and two in NSHD) symptoms declined in proportion. This led to a discrepancy in results between the binary and continuous outcomes, where the curves based on the continuous outcomes show a more positive profile: stable (NCDS) or declining (BCS70) psychological distress as opposed to increasing (both NCDS and BCS70). This also provides evidence for heterogeneity in in mid-life psychological distress, which future research could further examine.


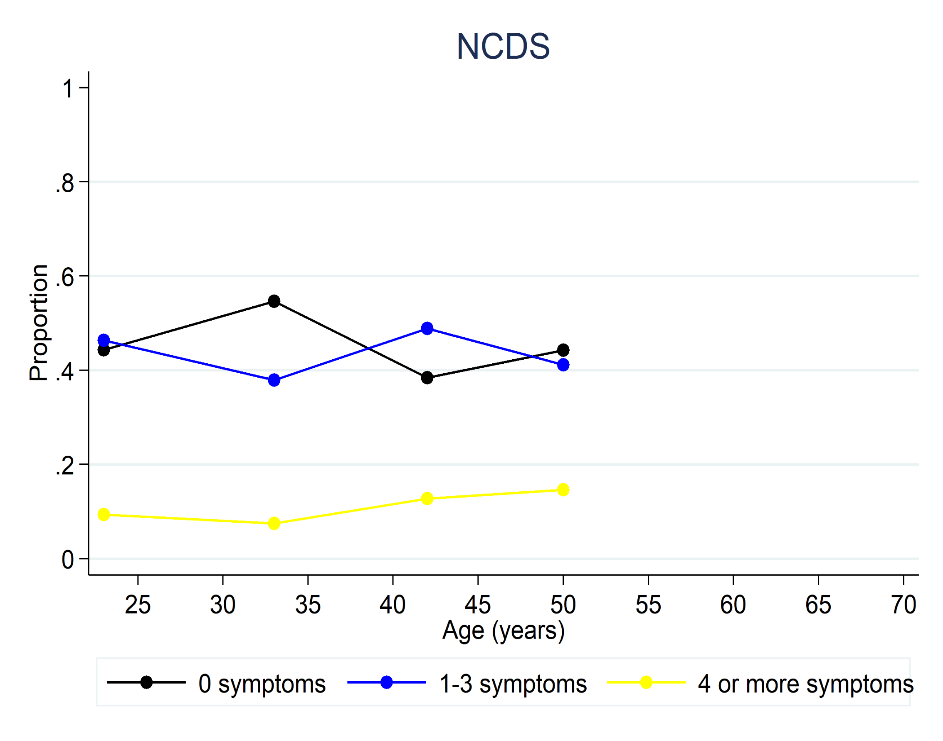

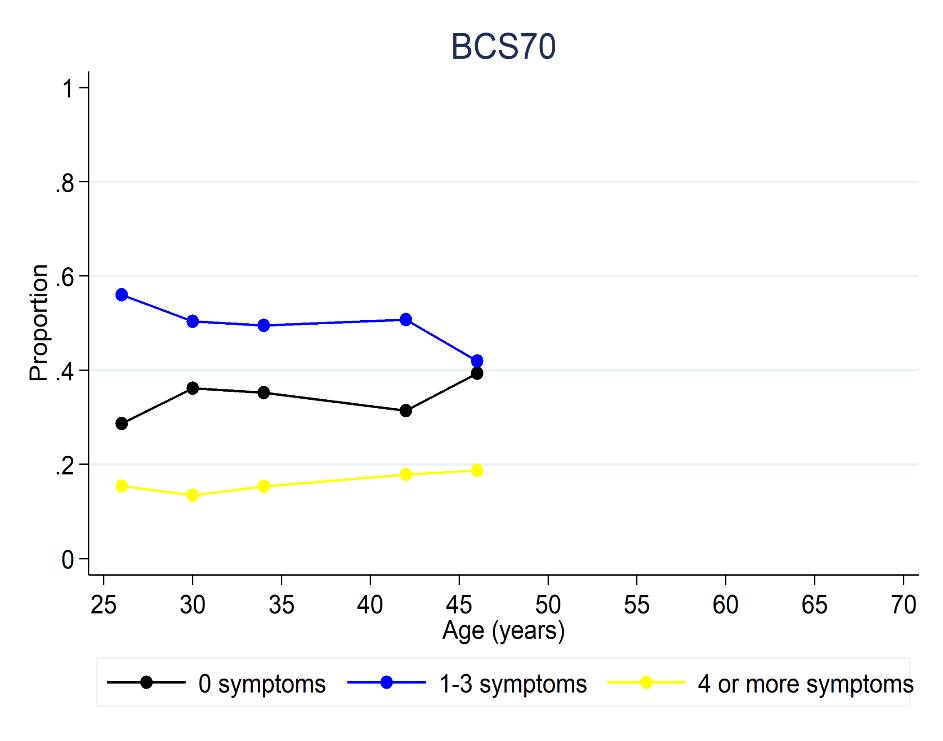

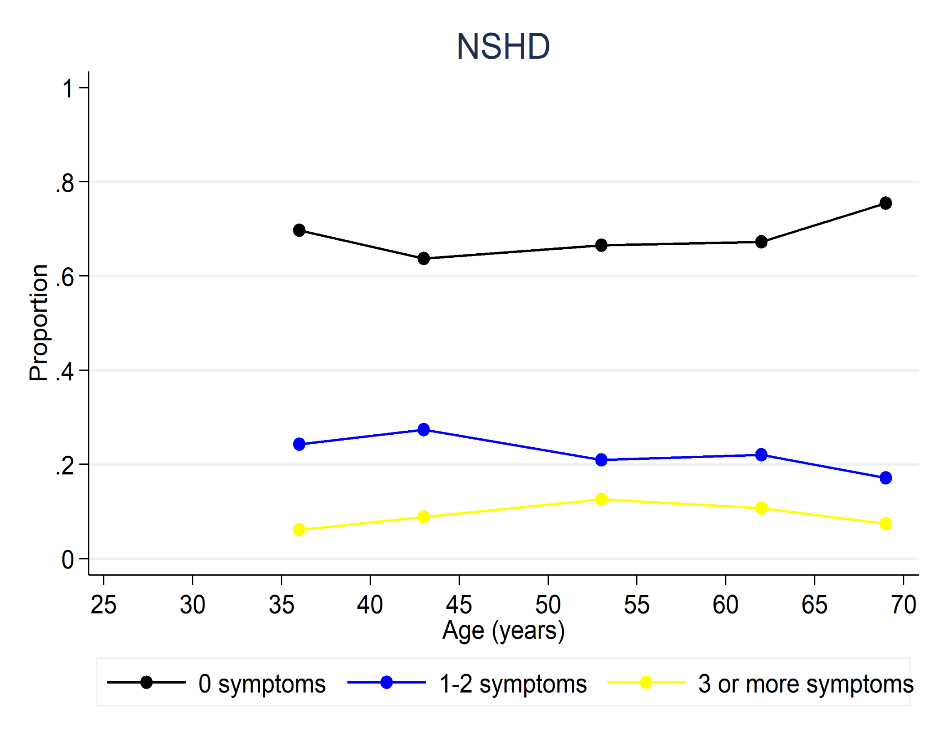


**PANEL A**

**PANEL B**

**PANEL C**

eFigure 2. Cohort-stratified age distribution of participants with varying proportions of symptoms.

eFigure 3. Gender-stratified age profile of psychological distress in the NSHD (1946 cohort), NCDS (1958 cohort), and BCS70 (1970 cohort)—estimates from multilevel logit regression based on the binary outcome.


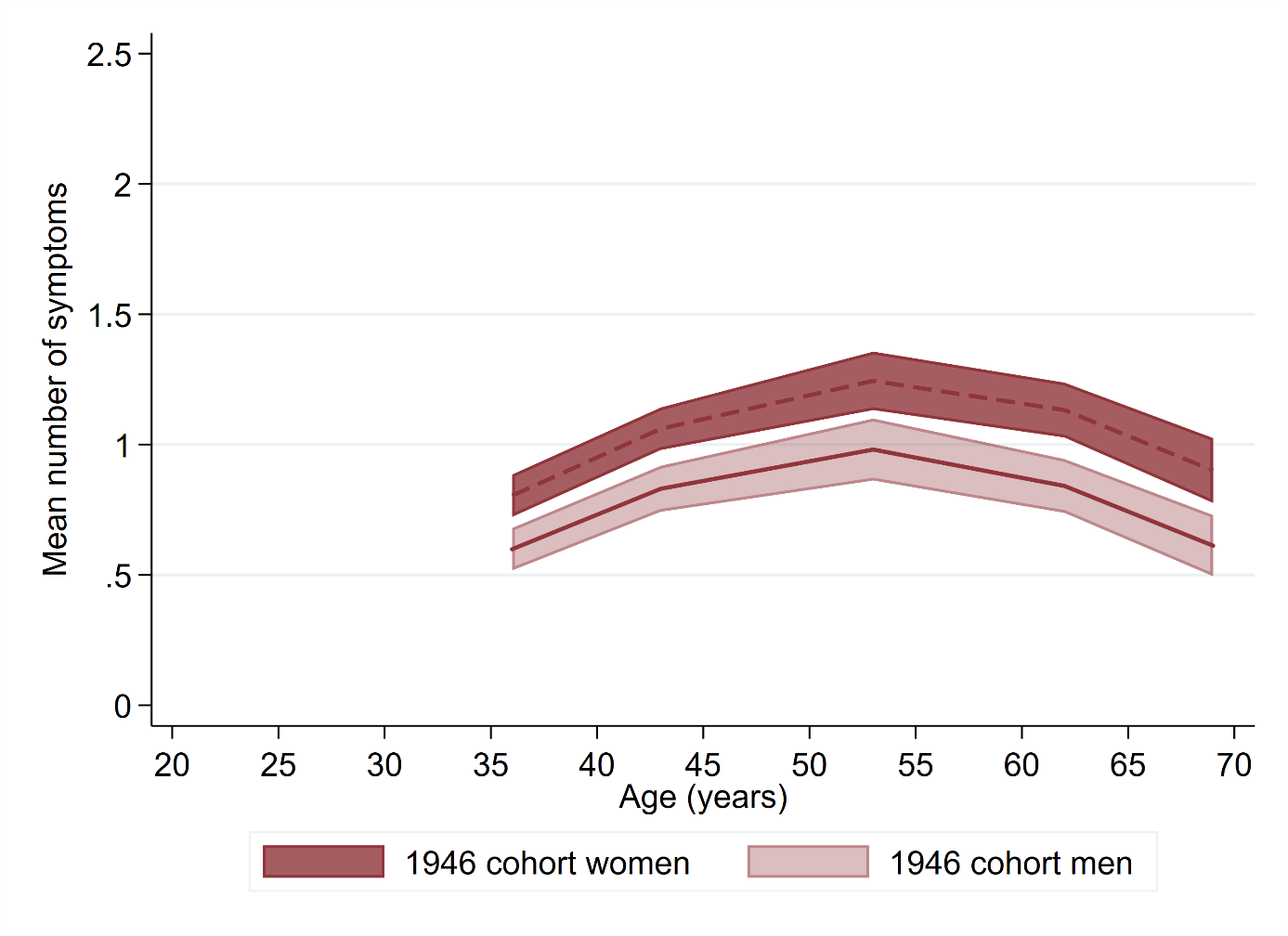


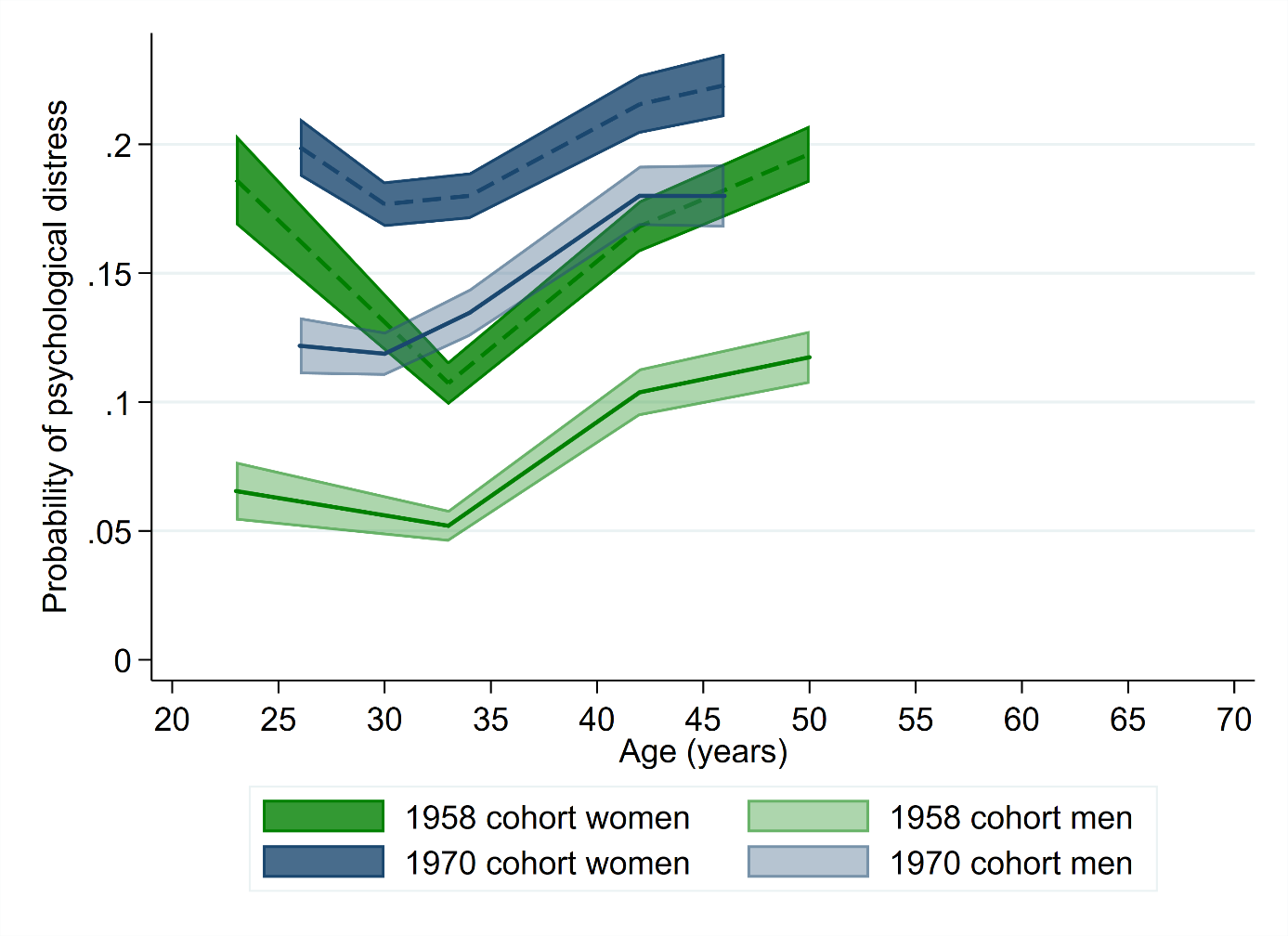


PANEL B


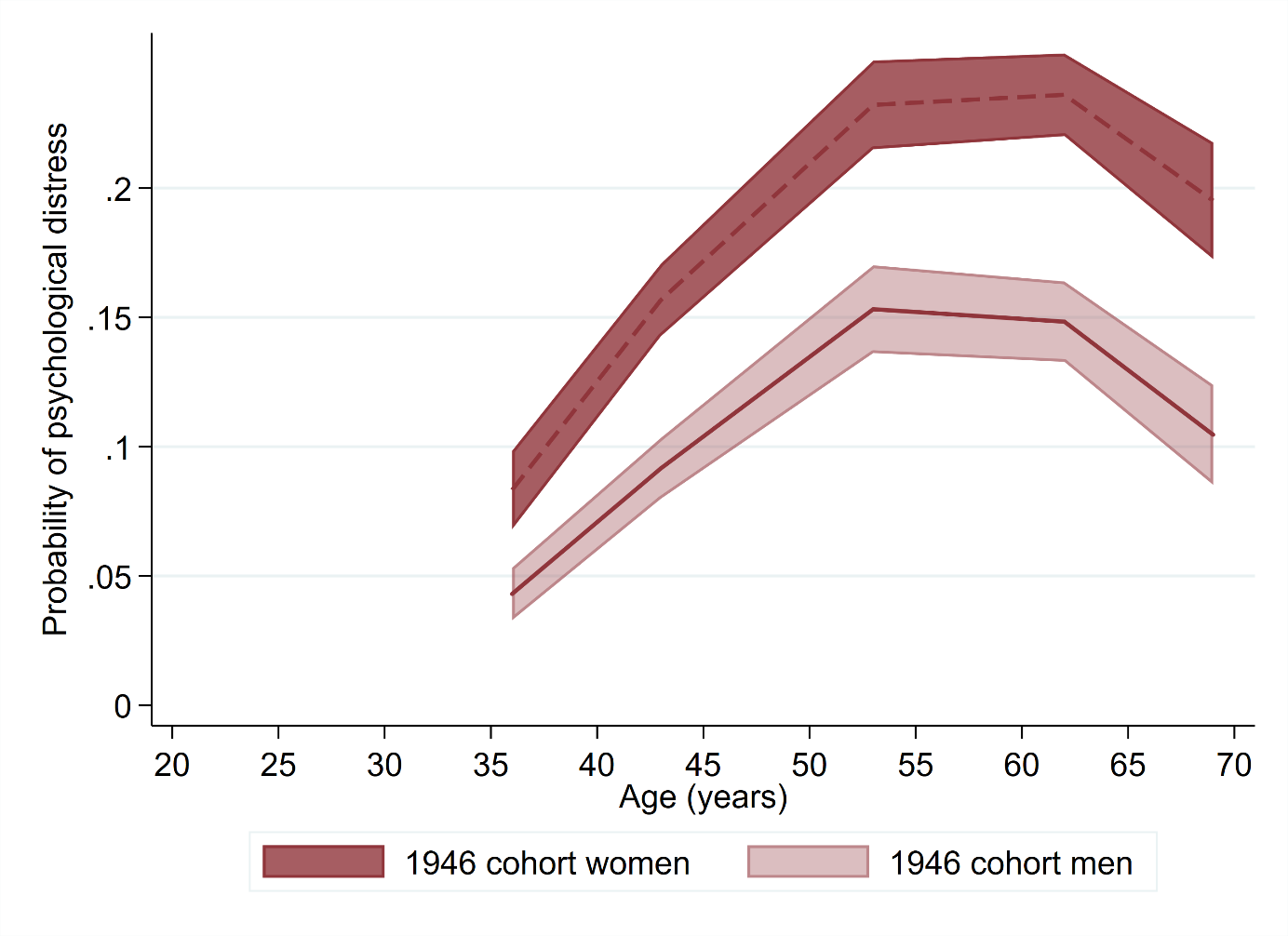


PANEL A

eFigure 4. Gender-stratified age profile of psychological distress in the NSHD (1946 cohort), NCDS (1958 cohort), and BCS70 (1970 cohort)—estimates from multilevel Poisson regression based on the continuous outcome.


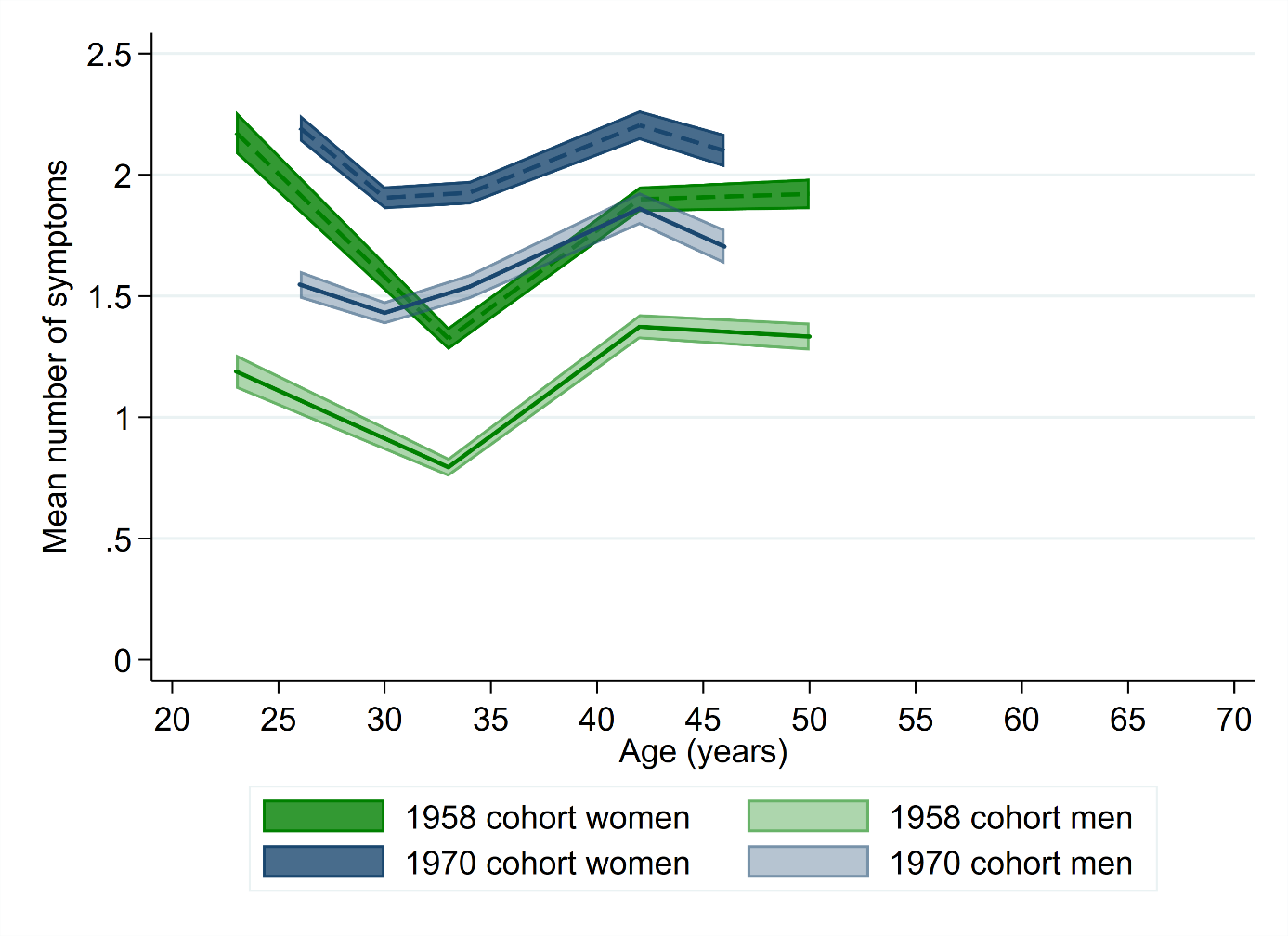

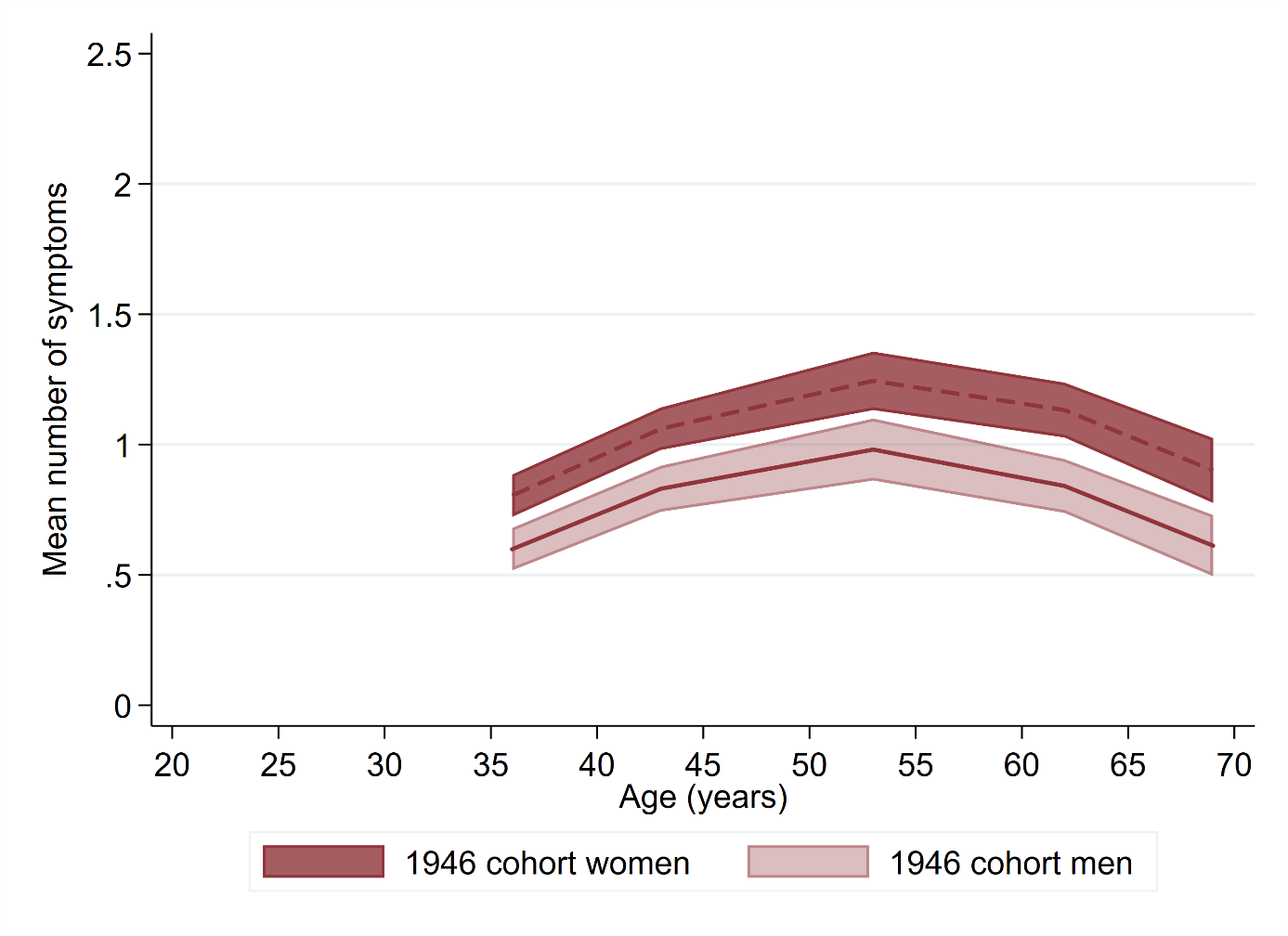


PANEL A

PANEL B
